# How Can We Balance Ethics and Law When Treating Smokers?

**DOI:** 10.5041/RMMJ.10238

**Published:** 2016-04-19

**Authors:** Helen Senderovich

**Affiliations:** Geriatrics & Pain Medicine & Palliative Care Physician, Baycrest Health Sciences, Toronto, ON, Canada; and Lecturer at the Department of Family and Community Medicine, Division of Palliative Care, University of Toronto, Toronto, ON, Canada

**Keywords:** Duty to treat, ethics, infectious, legal issues, physician-patient relationship, smokers

## Abstract

A physician is a valued member of society on whom many individuals rely for both professional advice and support during times when they may feel to be at a disadvantage, whether it be physically or mentally. An issue on the rise today concerns the population of smokers in our society. Many are coming to share the opinion that physicians should not provide treatments for smokers. Some of the opinions are based on the claim that smokers are morally responsible for their medical conditions. But, providing care in a fair manner includes not treating differently those who suffer from addiction. Moreover, it is important to recognize that allocating medical resources based on moral responsibility will undermine the physician–patient relationship which is necessary for the practice of medicine. Many countries have codes and policies that physicians must legally follow in terms of providing treatments. With acceptance of the fact that the patient may be unable to execute the decisions made by the physician, it is the legal duty of the physician to provide care and not abandon the patient. An analysis of the many policies around the world brings forward certain changes that must be made in order to make sure that physicians fulfil their legal duty, which is to provide care. As such, this article looks into the existing ethical dilemma in treating smokers around the world, with a review of some policies that will guide our approach in this matter.

## CASE EXAMPLE

Mrs X, a 61-year-old female patient in a long-term care facility, has been abusing tobacco since her teens. She has multiple comorbidities including a history of mental illness and chronic obstructive pulmonary disease (COPD). She also recently underwent emergency hernia repair. Her attempts to refrain from smoking resulted in an improvement of the healing of her wound, but it opened every time she relapsed. After much encouragement, Mrs X enrolled in a smoking cessation program and tried to follow the program’s regimen, but unfortunately yet again slipped back into a pattern of heavy smoking. Dr Y is becoming frustrated with the patient’s inability to commit to her treatment and is concerned about possible health complications secondary to her inability to stop smoking. He therefore decides to remove Mrs X from his practice and refer her to Dr Z.

## SCIENCE AND THE DILEMMA WITH TREATING SMOKERS

Smoking is a health risk to individuals, and it decreases the potential for benefit(s) from a variety of medical interventions. Extensive medical research has shown that nicotine is an addictive substance.^1^ Many surgical outcomes (e.g. cardiac, respiratory, prosthetic, spinal surgeries) including wound healing have demonstrated relatively improved results if patients stop smoking a few weeks before surgery. A study that examined the effects of smoking before a joint replacement surgery found that the probability of a smoker getting a wound infection was 3.3–3.4 times higher compared to that of a non-smoker.^2^ In addition, a faster reoccurrence of arteriosclerosis was observed in the smoking group, since the process of the arteries becoming blocked again was much faster in the smoking group compared to non-smokers.^3^ A clinical study of patients treated for thoracolumbar fractures showed that the risk of impaired bone healing has been estimated to be 3 to 18 times higher in smokers.^4^ Literature review states that patients should stop smoking 8 weeks prior to surgery in order to receive a benefit from it.^5^ Unfortunately, many patients are not able to comply with these recommendations by their physician.

It is important to note that some data also suggest that the relationship between smoking and surgical outcomes is equivocal, meaning some smokers who have surgery have no complications. In fact, a study that assessed patients who underwent arthroplasty of the hip and knee found that patients who smoked had fewer comorbidities than patients who did not smoke.^6^ Therefore, if we were to deny smokers access to surgical procedures, we are denying treatment to those patients who smoke, but who will not face any complications proceeding from treatment.

## TO TREAT, OR NOT TO TREAT, AROUND THE WORLD

The World Medical Association has established international ethical standards for the practice of medicine. One of its tasks was to compile the International Code of Medical Ethics and policy statements on specific ethical issues.

The doctor is instructed not to “permit considerations of age, disease or disability, creed, ethnic origin, gender, nationality, political affiliation, race, sexual orientation, social standing or any other factor to intervene between my duty and my patient.”^7^

Doctors must be loyal to their patients and offer them all the information regarding their procedures prior to treatment. If a proposed treatment is beyond a doctor’s scope of practice, he/she should refer the patient to another suitable physician.^7^ This is the decision that was made in the case of Mrs X, where Dr Y referred the patient to Dr Z, who agreed to provide the treatment.

### UK

Some doctors in England have refused to perform non-urgent coronary artery bypass surgery on smokers, requiring them to stop smoking first in order to be eligible for surgery. Surgeons have argued that non-smokers should be given preference over smokers for elective surgery because they will gain a greater benefit from it, and thus have a greater chance of complication-free survival.^8^ Underwood and Bailey state that “coronary bypass surgery should not be offered to smokers” due to the fact that smoking will increase the risk of postoperative complications, along with increasing the progression of coronary artery disease. Therefore, the benefits from the procedure will be reduced due to the resulting complications that may likely occur.^9^

Many also argued that smoking is self-inflicted, meaning that is was the patients’ choice, and thus they should not receive treatment.^10^ But, what many do not realize is that this leads to a slippery slope, such that overeaters, non-exercisers, terrorists who cause their own injury, drunk drivers, and non-compliant patients should not receive medical treatment. For example, if rugby players break their fingers from playing, do we refuse to treat them because they should have not taken the risk of playing? Providing a treatment should not be based on the assignment of blame.^11^ Moreover, it is important to recognize that people are entitled to make lifestyle choices, and thus we cannot deny or withhold treatment based on a choice that they have the right to make.^12^ The point is that all the patients entering a health care facility must be seen as equal, despite the difficulty in doing so. The duty of medical staff is to help towards maintaining and improving the patient’s health, and therefore a patient must not be judged while this duty is carried out. Therefore, making it legally permissible to refuse treatment for smokers will only lead to the refusal of treatment for those who are unmotivated, unfit, and/or seen as undeserving of treatment.^10^ The problem with this is: who is to determine what characteristics define someone as unmotivated and/or unfit? Ambiguity lies beneath these two terms. The licensing body of the UK states that a doctor must “take all possible steps to alleviate pain and distress whether or not a cure may be possible.”^13^ Therefore, patients should be advised and educated about the effects that smoking has on their health, but we cannot penalize them by denying the treatment that they require in order to alleviate any pain and/or distress. Moreover, the British Medical Association stated that physicians should not make decisions for their patients who do not refrain from smoking, by posing ultimatums, as they are legally not allowed to do this. Dr Graham Jackson, a consultant cardiologist at Guy’s Hospital and editor of the *British Journal of Clinical Practice*, had published a 10-year American study which stated that the survival rate of smokers undergoing by-pass surgery was 68%, whereas the survival rate for non-smokers was 84%.^14^ He stated: “The differences are not of sufficient scale to justify a ban on treating cigarette smokers.” David Blunkett, the Labour party’s health spokesman stated: “Everyone has a right to access to the National Health Services, no matter how foolish they have been in their own behaviour, whether that is in smoking or in fooling about in a boxing ring or on a rugby pitch.”^15^

### The Netherlands

In the Netherlands, there are laws in place which do not refuse treatment for smokers, regardless of the opinions of some doctors. One physician in the Netherlands wrote an article arguing that spending time on people who “willingly and knowingly damage their own and other’s health” was “wasted energy.”^3^ He felt that it was his professional duty to make it clear to his patients that smoking is dangerous and therefore should not be taken lightly. The government responded to his article by stating that it is unacceptable to exclude patients on the basis of their behavior. The government of Netherland states that, regardless of whether the treatment makes a difference in the patient’s health, it is the duty of the physician to provide the appropriate treatment and include a complement of supportive care.^16^ Patients cannot be discriminated against on the basis of their behavior, especially when it is a behavior that an individual has very little if any control over.^17^

### Australia

An editorial published in the *Medical Journal of Australia* states that smokers should not be offered a wide range of surgical procedures.^2^ The main argument was that smokers receive less benefit from treatment. As stated earlier, rates of wound infection are higher in smokers compared to non-smokers, and thus they also lead to delays in hospital discharge, and increased costs for hospital care.^5^ The issue of cost is prominent in countries such as Germany, France, the UK, and Italy, where over 73% of all EU27 health care spending was due to smoking.^18^ The caveat here is that denying patients treatment for reasons of saving on hospital costs may result in worse health spending down the road secondary to denying the procedure (e.g. expensive medications, repeat hospitalizations, etc.).^15^

The Royal Australasian College of Surgeons states that “Good medical practice involves … not prejudicing your patient’s care because you believe that a patient’s behavior has contributed to their condition.”^19^ This includes realizing that many treatments offer patients a potential psychological benefit. The patients may feel that they are attaining benefit from the treatment, and may therefore feel relief. As a result, though physicians are morally obligated to educate their patients about the effects of smoking, and advise them to stop before surgery, it is their legal responsibility to provide their patients with treatment, regardless of whether or not the patients cease their behavior.

### Canada

The College of Physicians and Surgeons of Ontario states that a physician must “act in patients’ best interests.” The document further states that physicians must “always be motivated by a regard for what is best for the patient.”^20^ These statements come to show that as long as the patient was informed about the risks associated with smoking, a physician must act in the patients’ best interests regardless of whether the patient may attain benefit from the treatment. Therefore, though Ontarians in Canada need to legally advise their patients about the risks and benefits of the treatment, they must respect their patients’ wishes.

## WHETHER TO ACCEPT THE RISK: PATIENTS WITH INFECTIOUS DISEASE VERSUS SMOKERS

The principle of respect and equality towards all patients has to be applied in the care of infectious patients. The World Medical Association (WMA) states that infectious patients should not be exempt from a physician’s duty to treat. In the case of acquired immune deficiency syndrome (AIDS), the WMA Interim Statement on AIDS, adopted in October 1987, states that “Patients with AIDS and those who test positively for the antibody to the AIDS virus must be provided with appropriate medical care and should not be treated unfairly or suffer from arbitrary or irrational discrimination in their daily lives.”^21^

Even the latest briefing note on Ebola virus disease issued by the World Health Organization discusses the rights, duties, and responsibilities of both workers and employers, emphasizing the necessity of adequate protective gear for health care staff. The necessity of adequate gear connects to the fact that all means are employed to ensure that health care workers are able to provide care for their patients.

Health care professionals who refuse to care for a patient without justification could suffer certain sanctions such as suspension from practice or a license revocation.^22^ Nevertheless, we do not have similar sanctions applied to health care professionals who refuse to treat smokers (unless the patient was abandoned by the health care professional).

The doctor’s principal obligation must be to the patient’s best interests, both in preventing and treating illness, and in helping the patient to cope with sickness or the nearing of death. Doctors may not refuse to help a patient because the patient has an infectious disease, and therefore health care providers must accept the risks that come with their line of work. If this is the case, why then should smokers be refused treatment? The risks associated with complications induced by smoking must be accepted as well, as in the case of Mrs X, since smokers should not be an exception to the rule by being denied treatment due to addiction.

## A DEEPER ANALYSIS OF ETHICS AND THE RELATIONSHIP BETWEEN ETHICS AND LAW

**Figure 1:**
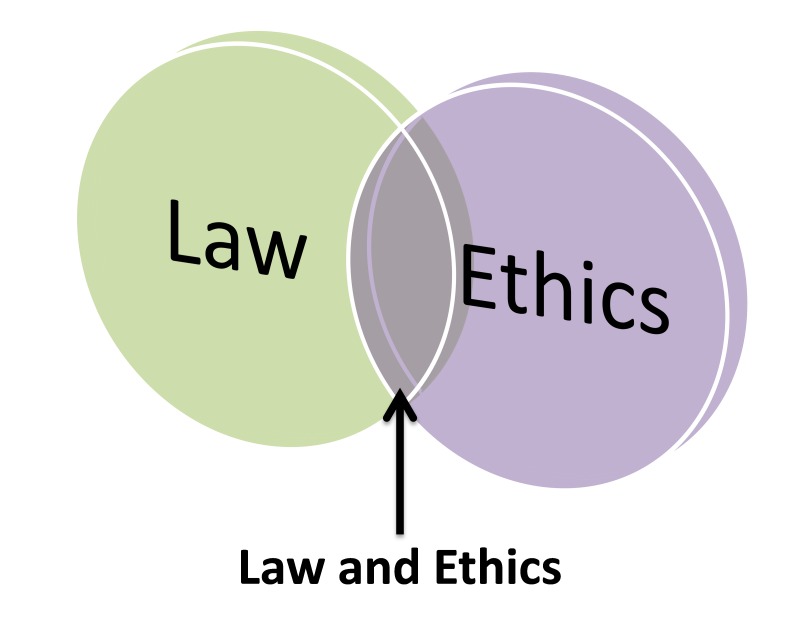
The Relationship Between Law and Ethics.

It is important to keep in mind that there is a close relationship between ethics and law (Figure 1). For example, page one of The Canadian Medical Association Code of Ethics states that a physician must “Provide appropriate care to the patient, even when cure is no longer possible, including physical comfort, and spiritual and psychosocial support.”^23^ This essentially states that even when a treatment shows no benefit to the patient, the physician must find ways to provide the patient with support and comfort. Furthermore, The American Medical Association states: “the social commitment of the physician is to sustain life and relieve suffering.”^23^ Therefore, a treatment which has the capacity to save life and/or relieve suffering should be provided. It is important to recognize that, many times, providing a treatment is what gives a patient comfort and possibly psychological benefit.

The WMA International Code of Medical Ethics states: “A physician shall act in the patient’s best interest when providing care.”^24^ Therefore, if we decide to treat smokers by first denying treatment in an effort to have them refrain from smoking, we may wonder if it is generally wise to subject smokers to such pain and public humiliation. Would we be acting in the patients’ best interests by subjecting them to pain and public humiliation? Can we afford simply to respect the “traditions” of others and agree to disagree? Mistaking no answer in practice for no answer in principle will lead us to conforming to the beliefs of the majority. What many do not realize is that physicians are legally not allowed to pose ultimatums for their patients who do not refrain from smoking, since the patient has the human right to autonomy. The Singapore Medical Council states that “A doctor shall not allow his personal beliefs to influence his management of his patients.”^25^ Therefore, despite the personal beliefs of physicians, in regard to patients who a smoke, physicians have the legal responsibility to care for their patients without discrimination.

## TREATING NON-COMPLIANT PATIENTS

Does our inability to have a certain policy in place, in regard to providing smokers with treatment, oblige us to respect all opinions equally? Of course not. In the same way, the fact that we may not be able to resolve specific dilemmas does not suggest that all competing responses to them are equally valid. Therefore, it is important to keep in mind the ethical and legal aspects of the doctor–patient relationship and the human rights of both individuals, in order to make a decision which will not be detrimental to the patient.

The WMA’s International Code of Medical Ethics states that there is only one reason for ending a physician–patient relationship: “Whenever an examination or treatment is beyond the physician’s capacity, he/she should consult with or refer to another physician who has the necessary ability.”^24^ They go on to state that if a physician decides to end the physician–patient relationship for any other reason, the physician must “be prepared to justify their decision, to themselves, to the patient and to a third party if appropriate.”^24^ As such, within the code itself, factors such as discrimination based on a patient’s behavior is not considered a valid reason for terminating the relationship. The Medical Board of Australia reinforces this statement by having a code that includes: “Not prejudicing your patient’s care because you believe that a patient’s behaviour has contributed to their condition.”^19^

The Medical Board of Australia states that physicians must discuss “with patients their condition and the available management options, including their potential benefit and harm.”^19^ The Board also states that physicians must be “recognizing and respecting patients’ rights to make their own decisions.”^19^ These two statements reinforce that a physician must act in the best interests of patients by recommending treatments that provide benefit. If a patient seeks treatment that contradicts the physician’s opinion, then the physician must ultimately respect the patients’ wishes. It is important to keep in mind that sometimes a treatment can provide both the patient and the family with benefits and thus achieve the aims of both the patient and the family. Therefore, a doctor cannot simply deny treatment to smokers due to the fact that it provides no physiological benefit, since the wishes of the patient and family may still be granted through the treatment.

In Manitoba (Canada) there are currently no guidelines or rules regarding refusal of care based on behaviors such as smoking. A Winnipeg doctor had told patients who smoke that they have to quit or find a new physician. Dr Bill Pope of the College of Physicians and Surgeons of Manitoba states: “It’s not uncommon for physicians to say under certain circumstances, I’m not prepared to treat certain kinds of patients.”^26^ Smokers are at risk for other complications after their treatments because they are not adhering to recommendations and physician advice, and thus do not quit smoking. Many times the lack of adherence is due to the fact that nicotine is addictive. Therefore, it is important to note that smoking cessation is not simply a non-adherence issue.

Physicians are required to advise their patients of the risks associated with smoking. If the physician does not notify the patient of the risks, he/she can be held liable if the patient develops complications associated with smoking.^16^ It is important to note that a physician can also be held liable if he/she refuse to treat a smoker due to the patient’s inability to follow the physician’s advice and refrain from smoking, when the treatment can still be beneficial.^17^ Hence, if Dr Y had decided no longer to provide treatment for Mrs X, since he felt that the constant treatments were non-beneficial due to her non-compliance regarding smoking, he should transfer her to another physician, such as Dr Z. If a physician decides to refuse to treat a patient, either due to the treatment being futile or the patient refusing to disengage from smoking, he/she must transfer the patient to another physician who is willing to accept the patient, in order not to be held liable. If another physician is not available, the physician should continue to provide treatment until a new physician can be located.^27^ Before transferring the patient, the physician must give the patient a Notice of Withdrawal of Services, so that the patient can find alternative medical care.^17^

## PROVIDING TRANSPLANTS TO SMOKERS

Organ transplant surgery is one of the procedures denied to smokers with the justification that there is increased morbidity and mortality after the surgery among smokers.^15^ It is believed that smoking in renal transplant patients is associated with increased graft failure, malignancies, and myocardial infarction.^28^ However, it is almost impossible to perform a randomized, controlled trial that could establish a better survival rate after organ transplantation in patients who quit smoking before surgery.^28^

We continue to face the ethical dilemma: do smokers deserve to be treated? We must discern between ethics and strong medical reasoning. The World Health Organization states: “donated organs should be made available to patients on the basis of medical need and not on the basis of financial or other consideration.”^29^ Thus, discrimination based on behavior is not considered a factor of allocation. For example, The Canadian Society of Transplantation prepares eligibility criteria for different transplant surgeries. It emphasizes that “Patients should be strongly encouraged to stop smoking before kidney transplantation. Patients who continue to smoke may be eligible for kidney transplantation with full informed consent regarding their increased risk.”^30^ The key word here is “may,” as the decision regarding who is to receive a transplant during times of low quantity is based on many factors including the patient’s “will to live, motivation and ability to follow post-operative directions.”^31^

This once again raises the question of whether or not a smoker can be held responsible for an addiction, since an addiction can make it hard for a smoker to follow post-operative directions. If the actions of psychotic, contagious, and chronically ill patients are perceived to be beyond their control and yet they are unquestioningly treated, then the actions of smokers, who are not adherent with the recommendation to refrain from smoking due to a loss of control associated with their addiction, should therefore be treated on the same basis as the other patients suffering from diverse illness(s) and multiple comorbidities.

The primary reason why individuals have argued against providing alcoholics a chance for a liver transplantation is because they feel that alcoholics are morally responsible for their alcohol-associated condition, and thus they should be placed lower on the priority list. We have heard a similar statement for smokers who are put lower on the priority list since they are said to be responsible for their smoking-associated condition. For example, alcoholic cirrhosis is a condition that is preventable by either abstaining from alcohol or using it in moderation. As such, about 85% of US liver transplant programs use the “6-month rule,” where patients are chosen for an organ transplant based on whether they can remain alcohol-free for 6 months.^32^ One of the purposes of this rule is to help the liver recover, and perhaps even avoid the transplant due to the liver healing by itself. The second is to see whether or not the individual is capable of being alcohol-free, since this will reduce the chances of relapse. Again, a similar restriction is placed on smokers, to reduce the risk of harm after treatment. But it is important to make sure that physicians are aware of the fact that the survival rates of patients with conditions related to alcohol use or smoking can be “at least as good as those seen with other indications.”^33^ Acknowledging this fact will improve patients’ access to liver and lung transplants, thus increasing the chances of saving a life.

Making decisions based on moral grounds also brings up the question of whether it is fair to refuse a transplant to a patient who had tried to overcome his/her addiction but, due to unfortunate circumstances, was not successful? For example, a patient who tries to overcome his/her addiction would seem more responsible than one who does not make efforts to quit. But then, what if a patient wanted to receive help to quit but was too poor to hire help and lived far from Alcoholics Anonymous or a smoking cessation program?^34^ How would we be able to decipher the responsible patients from the non-responsible patients? This poses a problem for this method of allocating medical resources. Moreover, basing the allocation of medical resources on what the patient verbally says may put the patient–physician relationship in jeopardy. The patient must feel comfortable in giving the relevant medical information to his/her physician and giving lifestyle information which is critical to determining an appropriate treatment and success rate. But, if the patient realizes that what he/she says may affect the chances of receiving a treatment such as an organ transplant, he/she will find every reason to withhold information that may put his/her treatment options in jeopardy.^34^ This now undermines the physician’s role as a health advocate and, furthermore, may not guarantee effective treatment for the patient. Therefore, allocating medical resources on the basis of moral responsibility will undermine two goals of the medical system—ensuring that patients have a medical safe harbor to turn to, and ensuring that they receive medical advocacy.^34^

## TREATING PATIENTS WITH BUERGER’S DISEASE

Thromboangiitis obliterans (TAO), or Buerger’s disease, is a non-atherosclerotic segmental vasculitis that affects the small and medium-sized arteries and veins of the extremities.^35^ Buerger’s disease is strongly associated with exposure to tobacco and thus is prevalent among smokers. Cannabis which is a co-factor of tobacco may also increase the risk of Buerger’s disease.^36^ Therefore, patients with Buerger’s disease are first and foremost told to quit smoking. Amputations are then recommended as a treatment option. It has been found that for those patients who do not stop smoking, most of the amputations occur due to relapses within the 6 years after diagnosis of Buerger’s disease.^37^ Moreover, smokers who smoked for more than 20 years were found to have a significant correlation with further major amputations.^37^ A study involving 27 cigarette smokers with Buerger’s disease found that all the smokers who reached cessation from smoking had improvement in the symptoms associated with their disease, and none of them had undergone amputation, compared to the 50% of patients who relapsed into smoking and needed an amputation.^38^ These results reinforce the fact that cessation from smoking improves symptoms of Buerger’s disease and reduces the likelihood of needing an amputation. Since cessation of smoking seems currently to be the one prominent solution, more treatment alternatives and programs need to be developed for individuals who are highly dependent on smoking. On the other hand, some studies have found that there is no significant difference in the limb salvage rate of smokers compared to ex-smokers. This implies that smoking cessation may not be advantageous to patients with Buerger’s disease.^39^ Regardless, it is important to recognize the fact that despite the patient’s addiction, physicians are still required to give their patients all the treatment options, while recommending practices which will help to reduce and alleviate the symptoms of the medical condition.

## IS IT APPROPRIATE TO TERMINATE THE PHYSICIAN–PATIENT RELATIONSHIP?

Patient abandonment takes place when a physician withdraws from caring for a patient and does not transfer his/her responsibilities to another physician who is qualified to provide care for the patient. This includes providing emergency treatment for the patient, even after the responsibilities have been handed over to another physician. Therefore, the previous physician still has the responsibility to provide care for the individual who was once his/her patient, in times of emergency, again showing that a physician cannot abandon his/her patient. Hence, if Mrs X suffered an emergency following the transfer of care to Dr Z, she is obliged to be treated by Dr Y. Overall, a physician has a legal duty to provide care to all patients regardless of their behavior and should therefore also have a valid reason before choosing to terminate a relationship which carries such value and importance to the patient.^27^

## CONCLUSION

This article aims to demonstrate that when discussing the refusal to treat smokers, we are actually bringing forward an interdependent world of facts, and thus science, ethics, and law should be taken into consideration when making decisions. It is therefore also important to keep in mind both science and ethics when making laws in health care.

“The interests of the patient should always be promoted regardless of financial arrangements; the health care setting; or patient characteristics, such as decision-making capacity, behavior, or social status.”^6^ Having said that, and in full knowledge of the fact that there is a duty to treat even when the risk for doctors’ health or potential liability is significant, is it ethical to deny access to health care to a group of patients such as smokers?

No it is not, and the answer is also found in the basic explanation of the human right to health care: “Health services, goods and facilities must be provided to all without any discrimination. Non-discrimination is a key principle in human rights and is crucial to the enjoyment of the right to the highest attainable standard of health.”^40^

With these Human Rights in place, society may continue to hold onto the fact that everyone will be cared for without discrimination, regardless of smoking behavior, which many have no control over. Physicians are valued members of society, and, as valued members, it is their legal duty to care for each individual to the best of their ability, and comply with the moral code which states that each individual has the right to health care.

## References

[1] Dullaart RP, Hoogenberg K, Dikkeschei BD, van Tol A (1994). Higher plasma lipid transfer protein activities and unfavorable lipoprotein changes in cigarette-smoking men. Arterioscler Thromb.

[2] Bikhchandani J, Varma SK, Henderson HP (2007). Is it justified to refuse breast reduction to smokers?. J Plast Reconstr Aesthet Surg.

[3] Centers for Disease Control and Prevention (US), National Center for Chronic Disease Prevention and Health Promotion (US); Office on Smoking and Health (US) (2010). How Tobacco Smoke Causes Disease: The Biology and Behavioral Basis for Smoking-Attributable Disease. A Report of the Surgeon General 2010.

[4] Canale ST, Kelly FB, Daugherty K (2012). Smoking threatens orthopaedic outcomes. American Academy of Orthopaedic Surgeons Now.

[5] Peters M (2007). Should smokers be refused surgery?. BMJ.

[6] (2008). The Right to Health. Fact Sheet No. 31. Office of the United Nations High Commissioner for Human Rights.

[7] World Medical Association (2015). Declaration of Geneva. The Handbook of WMA Policy.

[8] Bitomsky M (2012). Doctors propose surgery discrimination against smokers. Medical Post.

[9] Underwood M, Bailey J (1993). Coronary bypass surgery should not be offered to smokers. BMJ.

[10] Shiu M (1993). Refusing to treat smokers is unethical and a dangerous precedent. BMJ.

[11] Pawlik TM, Olver IN, Storm CD, Rodriguez MA (2009). Can physicians refuse treatment to patients who smoke?. J Oncol Pract.

[12] Lyons R (2012). The rationing of medical treatment is really sick. Spiked.

[13] Lavernia CJ, Sierra RJ, Gomez-Marin O (1999). Smoking and joint replacement: resource consumption and short-term outcome. Clin Orthop Relat Res.

[14] Jones J (1993). Doctors stand by refusal to treat smokers: Soapbox medicine or scientific medicine? Judy Jones reports on a divided profession. Independent.

[15] Scollo MM, Winstanley MH (2012). Tobacco in Australia: facts and issues.

[16] Jahring J (2007). Cessation of tobacco use. Tobacco Basics Handbook.

[17] World Medical Association (2009). Chapter Two: Physicians and Patients. Medical Ethics Manual.

[18] Sanco DG (2008). A study on liability and the health costs of smoking. Updated final report.

[19] Australian Medical Council (2014). Good medical practice: a code of conduct for doctors in Australia.

[20] The College of Physicians and Surgeons of Ontario (2011). Policy Statement #3–11: Complementary/alternative medicine. Dialogue.

[21] World Medical Association WMA statement on the professional responsibility of physicians in treating Aids patients.

[22] van der Weijden CP, Bredenoord AL, van Delden JJ (2010). The duty to treat in the context of an influenza pandemic. Vaccine.

[23] Canadian Medical Association (2015). CMA code of ethics. CMA Policy.

[24] Williams R (2015). Medical Ethics Manual.

[25] Singapore Medical Council (2012). Ethical code and ethical guidelines. All Healthcare Professionals 2012.

[26] Santalucia C, Michota FA (2004). When and how is it appropriate to terminate the physician-patient relationship?. Cleve Clin J Med.

[27] Kasiske BL, Klinger D (2000). Cigarette smoking in renal transplant recipients. J Am Soc Nephrol.

[28] Knoll G, Cockfield S, Blydt-Hansen T (2005). Canadian Society of Transplantation: Consensus guidelines on eligibility for kidney transplantation. CMAJ.

[29] Touraine JL, Traeger J, Bétuel H, Dubernard JM, Revillard JP, Dupuy C (1998).

[30] Abouna GM (2003). Ethical issues in organ and tissue transplantation. Exp Clin Transplant.

[31] Sagan A (2015). Organ donation ethics: how doctors decide who gets a transplant. CBCNews.

[32] Bramstedt K, Jabbour N (2006). When alcohol abstinence criteria create ethical dilemmas for the liver transplant team. J Med Ethics.

[33] Perut V, Conti F, Scatton O, Soubrane O, Calmus Y, Vidal-Trecan G (2009). Might physicians be restricting access to liver transplantation for patients with alcoholic liver disease?. J Hepatol.

[34] Ho D (2008). When good organs go to bad people. Bioethics.

[35] Arkkila P (2006). Thromboangiitis obliterans (Buerger’s disease). Orphanet J Rare Dis.

[36] Martin-Blondel G, Koskas F, Cacoub P, Sène D (2011). Is thromboangiitis obliterans presentation influenced by cannabis addiction?. Ann Vasc Surg.

[37] Fazeli B, Ravari H, Assadi R (2012). Natural history definition and a suggested clinical approach to Buerger’s disease: a case-control study with survival analysis. Vascular.

[38] Jiménez-Ruiz CA, Dale LC, Astray Mochales J, Velázquez Buendía L, de Granda Orive I, Guirao García A (2006). Smoking characteristics and cessation in patients with thromboangiitis obliterans. Monaldi Arch Chest Dis.

[39] Sugimoto M, Miyachi H, Morimae H (2015). Fate of ischemic limbs in patients with Buerger’s disease based on our 30-year experience: does smoking have a definitive impact on the late loss of limbs?. Surg Today.

[40] Reid JL, Hammond D, Burkhalter R, Ahmed R Tobacco Use in Canada: Patterns and Trends.

